# Clinical characteristics and survival outcomes of patients with pneumonectomies: A population-based study

**DOI:** 10.3389/fsurg.2022.948026

**Published:** 2022-08-09

**Authors:** Linlin Wang, Lihui Ge, Guofeng Zhang, Ziyi Wang, Yongyu Liu, Yi Ren

**Affiliations:** ^1^ Department of Thoracic Surgery, Shenyang Chest Hospital & Tenth People's Hospital, Shenyang, China; ^2^ Department of Health Management, Shengjing Hospital of China Medical University, Shenyang, China

**Keywords:** pneumonectomy, SEER database, non-small cell lung cancer, propensity score matching, forest plot

## Abstract

**Background:**

Prognostic factors in a pneumonectomy (PN) are not yet fully defined. This study sought to analyze and evaluate long-term survival after pneumonectomies (PNs) for patients with non-small cell lung cancer (NSCLC).

**Methods:**

We obtained data from the Surveillance, Epidemiology, and End Results (SEER) database for patients who underwent PNs between 2004 and 2015. Propensity score matching (PSM) analysis and Kaplan–Meier curves were used to estimate overall survival (OS), while univariate and multivariable Cox proportional hazards regression analyses were applied to create a forest plot.

**Results:**

In total, 1,376 patients were grouped according to right/left PNs. Before matching, OS was worse after a right PN [hazard ratio (HR): 1.459; 95% CI 1.254–1.697; *P* < 0.001] and after matching, survival differences between groups were not significant (HR: 1.060; 95% CI 0.906–1.240; *P* = 0.465). Regression analysis revealed that age, gender, grade, lymph node dissection, N-stage, and chemotherapy were independent predictors of OS (*P* < 0.05). Chemotherapy was associated with improved OS (*P* < 0.001).

**Conclusions:**

Laterality was not a significant prognostic factor for long-term survival after a PN for NSCLC. Chemotherapy was a significant independent predictor of improved OS. Long-term survival and outcomes analyses should be conducted on larger numbers of patients.

## Introduction

The incidence of lung cancer and associated mortality rates are among the highest of all malignant tumors in China and the world. The most recent estimate predicts 236,740 new cases and 130,180 deaths in 2022, emphasizing the serious worldwide effects of this disease, which has a 5-year survival rate of approximately 22 percent (1). About 80% of patients with lung cancer have NSCLC. Radical resection remains the preferred treatment for NSCLC and current widely-accepted surgical techniques include lobectomy, segmentectomy, PN, and pulmonary sleeve with pulmonary artery reconstruction (2–4).

Central lung tumors are relatively common in clinical practice. Some can be treated with lobectomy or pulmonary sleeve resection, but when these methods cannot remove the tumor completely, a PN is required, including the dissection of half of the lung tissue and the pulmonary arteries, veins, and main bronchi, accompanied by systematic lymph node dissection. The prognostic factors for left and right PNs remain under investigation. Dr. Graham reported the world's first PN for lung cancer in 1933 (5). However, a PN remains challenging with high complications, such as bronchopleural fistula, progressive pulmonary hypertension, respiratory failure, etc. (6, 7). Depending on the surgeon's experience, and the histological and anatomical characteristics of the lung and tumor, survival may be better for a left PN than the right (8). The reason may be that the contribution of the right lung to the whole is larger than that of the left lung (9).

It is controversial whether the treatment of lung cancer patients with left or right PN is beneficial to long-term survival. And survival data is lacking in the existing literature (10). This study aimed to analyze and evaluate long-term survival after a PN in patients with NSCLC. We used a population-based national registry, the SEER database, to analyze clinical characteristics and prognosis for unilateral and bilateral PNs. We analyzed the factors affecting PNs and applied the Cox proportional hazards model to create a forest plot of individual hazard ratios for the overall survival of patients who underwent left vs. right PNs.

## Materials and methods

### Patients

**Figure 1:**
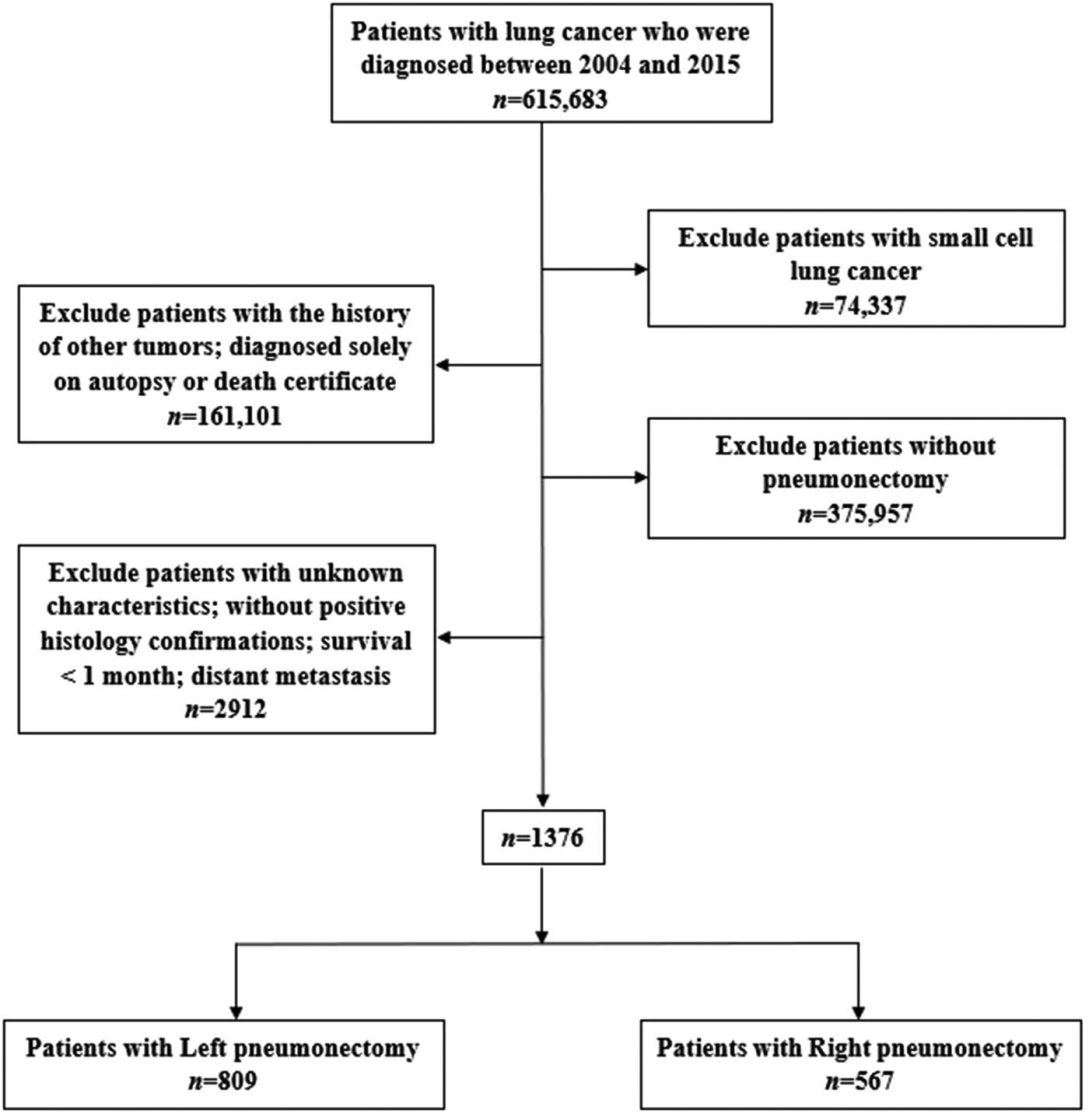
Flow chart showing patient selection.

We extracted data from the SEER database (https://seer.cancer.gov/) through SEER*Stat software (v8.3.6, https://seer.cancer.gov/seerstat/) to identify patients with a confirmed diagnosis of NSCLC between 2004 and 2015 and those who underwent a PN (SEER Surgery Codes: 55, 56, 65, 66, 70) were included in our study. The inclusion criteria were: (1) diagnosis between 2004 and 2015 (615,683), (2) diagnosis of NSCLC confirmed microscopically, (3) only one primary tumor and available clinical information, (4) survival for at least 1 month and active follow-up. The exclusion criteria were: (1) incomplete survival or clinical data, including unknown race, grade, etc.; distant metastasis; survival for <1 month (2864), (2) small cell lung cancer (74,337), (3) without pneumonectomy (375,957), (4) with the history of other tumors and diagnosed solely on autopsy or death certificate (161,101; Figure 1).

### Variables

The covariates included age, gender, race, marriage, primary site, summary stage, grade, lymph node dissection, tumor stage, T-stage, N-stage, radiotherapy, and chemotherapy. We classified age into four groups: ≤50, 51–60, 61–70, and 71 and older. The grade was classified as well-differentiated (I), moderately differentiated (II), poorly differentiated (III), and undifferentiated (IV). The lymph node dissections included 1–3 removed and ≥4 removed. We followed the eighth edition American Joint Committee on Cancer (AJCC) lung cancer staging system and updated the T-stage (T1, T2, T3, and T4), N-stage (N0, N1, N2–N3), and tumor stage (I, II, III) for all patients in all periods. Overall survival (OS) was defined as the time from diagnosis to death from any cause. To avoid bias between left and right PN groups, we applied 1: 1 PSM (11) for age, gender, race, marriage, primary site, grade, summary stage, lymph node dissection, tumor stage, T-stage, N-stage, radiotherapy, and chemotherapy.

### Statistical analysis

Continuous variables are expressed as mean ± SD and categorical variables are expressed as percentages. Variables were conducted by Student's *t* test, Pearson's Chi-square test, and ANOVA. Kaplan–Meier method we used to create survival curves and the differences between the curves were analyzed using a log-rank test. The Cox proportional hazards model was used to verify independent prognostic factors and calculate the HR and corresponding 95% CI. Forest plots describes specific results. We used the Statistical Product and Service Solutions 25.0 software (SPSS, Inc., Chicago, IL, United States) to analyze data. *P* values <0.05 (two-sided) were considered statistically significant. The forest plot and survival curves were drawn with a GraphPad Prism (Version 8.3.1).

## Results

### Patient characteristics

A total of 1,376 patients who underwent a PN between 2004 and 2015 were selected from the SEER database in this study. Of these, 809 (58.79%) had a left PN and 567 (41.21%) had a right PN. The primary tumor site was significantly different between the left- and right-sided PN groups (*P* < 0.05). The patient characteristics are shown in Table 1.

**Table 1:** Baseline patient characteristics before and after matching.

Characteristic	Pneumonectomy unmatching				Pneumonectomy matching			
	Total	Left side	Right side	*P*^b^ value	Total	Left side	Right side	*P* value
	*N* = 1376	*N* = 809	*N* = 567		*N* = 1100	*N* = 550	*N* = 550	
**Age (years), *n* (%)**				0.339				0.598
≤50	186 (13.5)	100 (12.4)	86 (15.2)		163 (14.8)	83 (15.1)	80 (14.5)	
51–60	427 (31.0)	253 (31.3)	174 (30.7)		341 (31.0)	171 (31.1)	170 (30.9)	
61–70	494 (35.9)	302 (37.3)	192 (33.9)		387 (35.2)	200 (36.4)	187 (34.0)	
≥71	269 (19.5)	154 (19.0)	115 (20.3)		209 (19.0)	96 (17.5)	113 (20.5)	
Mean ± SD	61.44 ± 10.91	61.60 ± 10.61	61.21 ± 11.34	0.519	61.14 ± 10.95	60.91 ± 10.74	61.38 ± 11.15	0.478
**Gender, *n* (%)**				0.869				0.850
Female	501 (36.4)	296 (36.6)	205 (36.2)		395 (35.9)	196 (35.6)	199 (36.2)	
Male	875 (63.6)	513 (63.4)	362 (63.8)		705 (64.1)	354 (64.4)	351 (63.8)	
**Race, *n* (%)**				0.574				0.731
White	1,187 (86.3)	696 (86.0)	491 (86.6)		959 (87.2)	483 (87.8)	476 (86.5)	
Black	111 (8.1)	63 (7.8)	48 (8.5)		85 (7.7)	39 (7.1)	46 (8.4)	
Others	78 (5.7)	50 (6.2)	28 (4.9)		56 (5.1)	28 (5.1)	28 (5.1)	
**Marriage, *n* (%)**				0.758				0.951
No^a^	566 (41.1)	330 (40.8)	236 (41.6)		459 (41.7)	229 (41.6)	230 (41.8)	
Yes	810 (58.9)	479 (59.2)	331 (58.4)		641 (58.3)	321 (58.4)	320 (58.2)	
**Primary site, *n* (%)**				0.003				0.090
Main bronchus	129 (9.4)	79 (9.8)	50 (8.8)		80 (7.3)	30 (5.5)	50 (9.1)	
Lobe^c^	1,067 (77.5)	647 (80.0)	420 (74.1)		866 (78.7)	446 (81.1)	420 (76.4)	
Overlapping lesion of lung	120 (8.7)	56 (6.9)	64 (11.3)		102 (9.3)	47 (8.5)	55 (10.0)	
Lung NOS	60 (4.4)	27 (3.3)	33 (5.8)		52 (4.7)	27 (4.9)	25 (4.7)	
**Summary stage, *n* (%)**				0.394				0.777
Localized	224 (16.3)	129 (15.9)	95 (16.8)		179 (16.3)	87 (15.8)	92 (16.7)	
Regional	1,027 (74.6)	613 (75.8)	414 (7301)		818 (74.4)	414 (75.3)	404 (73.5)	
Distant	125 (9.1)	67 (8.3)	58 (10.2)		103 (9.4)	49 (8.9)	54 (9.8)	
**Grade** ^**d**^ **, *n* (%)**				0.916				0.634
I	119 (8.6)	72 (8.9)	47 (8.3)		95 (8.6)	53 (9.6)	42 (7.6)	
II	530 (38.5)	314 (38.8)	216 (38.1)		413 (37.5)	200 (36.4)	213 (38.7)	
III	685 (549.8)	400 (49.4)	285 (50.3)		557 (50.6)	279 (50.7)	278 (50.5)	
IV	42 (3.1)	23 (2.8)	19 (3.4)		35 (3.2)	18 (3.3)	17 (3.1)	
**Lymph node dissection, *n* (%)**				0.561				0.753
1–3 removed	44 (3.2)	24 (3.0)	20 (3.5)		42 (3.8)	22 (4.0)	20 (3.6)	
≥4 removed	1,332 (96.8)	785 (97.0)	547 (96.5)		1,058 (96.2)	528 (96.0)	530 (96.4)	
**Tumor stage (AJCC 8th ed.), *n* (%)**				0.100				0.419
I	169 (12.3)	95 (11.7)	74 (13.1)		129 (11.7)	58 (10.5)	71 (12.9)	
II	377 (27.4)	239 (29.5)	138 (24.3)		285 (25.9)	148 (26.9)	137 (24.9)	
III	830 (60.3)	475 (58.7)	355 (62.6)		686 (62.4)	344 (62.5)	342 (62.2)	
**T-stage, *n* (%)**				0.320				0.717
T1	143 (10.4)	83 (10.3)	60 (10.6)		111 (10.1)	53 (9.6)	58 (10.5)	
T2	403 (29.3)	252 (31.1)	151 (26.6)		305 (27.7)	155 (28.2)	150 (27.3)	
T3	322 (23.4)	181 (22.4)	141 (24.9)		263 (23.9)	125 (22.7)	138 (25.1)	
T4	508 (36.9)	293 (36.2)	215 (37.9)		421 (38.3)	217 (39.5)	204 (37.1)	
**N-stage, *n* (%)**				0.628				0.850
N0	511 (37.1)	298 (36.8)	216 (37.6)		412 (37.5)	208 (37.8)	204 (37.1)	
N1	546 (39.7)	329 (40.7)	217 (38.3)		419 (38.1)	205 (37.3)	214 (38.9)	
N2–3	319 (23.2)	182 (22.5)	137 (24.2)		269 (24.5)	137 (24.9)	132 (24.0)	
**Radiotherapy, *n* (%)**				0.855				0.553
No/unknown	1,105 (80.3)	651 (80.5)	454 (80.1)		870 (79.1)	431 (78.4)	439 (79.8)	
Yes	271 (19.7)	158 (19.5)	113 (19.9)		230 (20.9)	119 (21.6)	111 (20.2)	
**Chemotherapy, *n* (%)**				0.170				0.629
No/Unknown	625 (45.4)	355 (43.9)	270 (47.4)		520 (47.3)	264 (48.0)	256 (46.5)	
Yes	751 (54.6)	454 (56.1)	297 (52.4)		580 (52.7)	286 (52.0)	294 (53.5)	
**OS** ^**e**^ **, *n* (%)**				<0.001				0.100
Alive	677 (49.2)	361 (44.6)	316 (55.7)		471 (42.8)	222 (40.4)	249 (45.3)	
Dead	699 (50.8)	448 (55.4)	251 (44.3)		629 (57.2)	328 (59.6)	301 (54.7)	

^a^ Includes separated, single (never married), divorced, unmarried or domestic partners, unknown and widowed.

^b^ *P* value between left and right PNs was calculated by chi-square test, respectively.

^c^ Includes upper lobe, middle lobe and lower lobe.

^d^ Grade was classified as well-differentiated (I), moderately differentiated (II), poorly differentiated (III), and undifferentiated (IV).

^e^ OS, overall survival.

### Survival analysis

**Figure 2:**
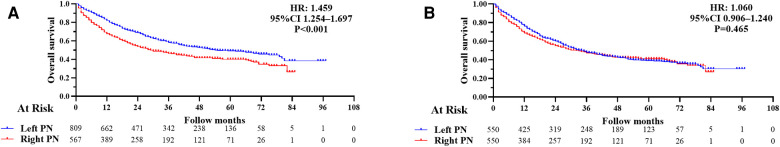
(**A**) Kaplan–Meier survival curves for overall survival following a left and right pneumonectomy (PN) before propensity score matching (overall survival, hazard ratio (HR): 1.459; 95% CI 1.254–1.697; *P* < 0.001). (**B**) Kaplan–Meier survival curves for overall survival in left and right PN after propensity score matching (overall survival, HR: 1.060; 95% CI 0.906–1.240; *P* = 0.465).

The mean follow-up in all 1,376 patients was 31.26 ± 23.04 months (33.65 ± 23.01 months for a left PN and 28.85 ± 22.68 months for a right PN). Median OS was 54 (95% CI 41.62–66.38) months for a left PN vs. 29 (95% CI 22.01–36.00) months for a right PN. One-, three-, and five-year overall survival rates for all patients, left and right PN patients were 74.6%, 42.8%, and 22.3%; 82.0%, 58.4%, and 49.0%; and 67.6%, 46.3%, and 39.3%, respectively. Kaplan–Meier survival analysis suggested that OS was significantly worse for patients who had a right PN (HR: 1.459; 95% CI 1.254–1.697; *P* < 0.001) compared with a left PN (Figure 2A).

**Figure 3:**
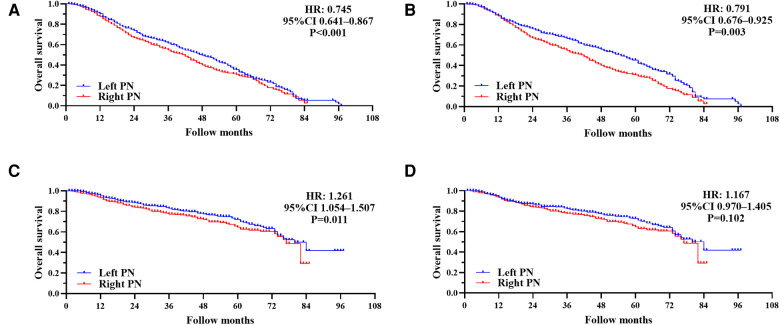
(Top row) Kaplan–Meier survival curves for chemotherapy in patients with non-small cell lung cancer (NSCLC) after a PN. (**A**) Before propensity score matching (PSM), HR: 0.745; 95% CI 0.641–0.867; *P* < 0.001; (**B**) after PSM, HR: 0.791; 95% CI 0.676–0.925; *P* = 0.003. (Bottom row) Kaplan–Meier survival curves for radiotherapy in NSCLC patients after PN. (**C**) Before PSM, HR: 1.261; 95% CI 1.054–1.507; *P* = 0.011; (**D**) after PSM, HR: 1.167; 95% CI 0.970–1.405; *P* = 0.102.

We used univariate analysis to identify possible prognostic factors in PNs for NSCLC and found statistically significant (*P* < 0.05) correlations between OS and laterality, age, gender, summary stage, grade, tumor stage, T-stage, N-stage, lymph node dissection, radiotherapy, and chemotherapy (Table 2). Chemotherapy was associated with a better prognosis and radiotherapy was associated with a worse prognosis (Figures 3A,C). Race, marriage, and primary site were not significant prognostic factors in our univariate analysis (*P* > 0.05). Compared with right PNs, the characteristics of those with left PNs were as follows: age ≤50 years (*P* = 0.004), 61–70 years (*P* < 0.001); female (*P* = 0.009), male (*P* < 0.001); white (*P* < 0.001); unmarried (*P* = 0.004), married (*P* < 0.001); single lobe (*P* < 0.001) and overlapping lesion (*P* = 0.003); II (*P* = 0.002), III (*P* < 0.001) and IV (*P* = 0.027); localized (*P* = 0.018) and regional (*P* < 0.001); number of regional lymph nodes dissected ≥4 (*P* < 0.001); tumor stage II (*P* < 0.001) and III (*P* = 0.002); T2 (*P* = 0.005) and T4 (*P* = 0.001); N0 (*P* < 0.001) and N1 (*P* < 0.001); no/unknown radiotherapy (*P* < 0.001); no/unknown chemotherapy (*P* < 0.001) and chemotherapy (*P* = 0.006).

**Table 2:** Univariate and multivariable analysis of OS^a^ before and after matching.

Characteristic	Unmatching				Matching			
	Univariate analysis		Multivariable analysis		Univariate analysis		Multivariable analysis	
	HR^b^ (95% CI^c^)	*P* value	HR (95% CI)	*P* value	HR (95% CI)	*P* value	HR (95% CI)	*P* value
**Laterality**								
Left	Reference		Reference		Reference			
Right	1.459 (1.254–1.697)	<0.001	1.480 (1.271–1.725)	<0.001	1.060 (0.906–1.240)	0.465		
**Age (years)**								
≤50	Reference		Reference		Reference		Reference	
51–60	1.249 (0.955–1.633)	0.104	1.254 (0.954–1.650)	0.105	1.396 (1.060–1.839)	0.018	1.357 (1.025–1.796)	0.033
61–70	1.500 (1.157–1.945)	0.002	1.315 (1.007–1.718)	0.044	1.728 (1.322–2.257)	<0.001	1.450 (1.103–1.905)	0.008
≥71	2.008 (1.524–2.646)	<0.001	1.739 (1.306–2.317)	<0.001	2.254 (1.693–3.002)	<0.001	1.890 (1.406–2.540)	<0.001
**Gender**								
Female	Reference		Reference		Reference		Reference	
Male	1.410 (1.197–1.661)	<0.001	1.295 (1.097–1.528)	0.002	1.382 (1.167–1.637)	<0.001	1.262 (1.063–1.498)	0.008
**Race**								
White	Reference				Reference			
Black	1.022 (0.775–1.327)	0.878			1.027 (0.769–1.372)	0.855		
Others	1.019 (0.734–1.415)	0.912			1.061 (0.746–1.508)	0.742		
**Marriage**								
No^d^	Reference				Reference			
Yes	0.879 (0.755–1.024)	0.098			0.893 (0.763–1.046)	0.161		
**Primary site**								
Main bronchus	Reference				Reference			
Lobe	1.174 (0.883–1.560)	0.271			0.908 (0.669–1.231)	0.533		
Overlapping lesion of lung	1.295 (0.896–1.872)	0.170			0.811 (0.545–1.207)	0.302		
Lung NOS	1.609 (1.040–2.489)	0.033			0.940 (0.583–1.515)	0.798		
**Summary stage**								
Localized	Reference		Reference		Reference		Reference	
Regional	1.761 (1.382–2.243)	<0.001	1.385 (1.016–1.889)	0.040	1.864 (1.449–2.399)	<0.001	1.369 (0.996–1.883)	0.053
Distant	2.306 (1.672–3.181)	<0.001	1.478 (1.001–2.183)	0.050	2.304 (1.645–3.227)	<0.001	1.429 (0.953–2.143)	0.084
**Grade** ^**e**^								
I	Reference		Reference		Reference		Reference	
II	1.977 (1.368–2.856)	<0.001	1.705 (1.159–2.508)	0.007	2.293 (1.549–3.394)	<0.001	1.830 (1.216–2.752)	0.004
III	2.537 (1.768–3.641)	<0.001	2.062 (1.410–3.017)	<0.001	2.743 (1.866–4.032)	<0.001	2.069 (1.382–3.097)	<0.001
IV	2.710 (1.585–4.631)	<0.001	2.583 (1.487–4.488)	0.001	2.782 (1.579–4.900)	<0.001	2.416 (1.348–4.333)	0.003
**Lymph node dissection**								
1–3 removed	Reference		Reference		Reference		Reference	
≥4 removed	0.589 (0.408–0.850)	0.005	0.555 (0.382–0.805)	0.002	0.648 (0.449–0.936)	0.021	0.599 (0.412–0.870)	0.007
**Tumor stage (AJCC 8th ed.)**								
I	Reference		Reference		Reference		Reference	
II	1.181 (0.864–1.615)	0.298	0.978 (0.676–1.416)	0.907	1.288 (0.929–1.785)	0.128	0.970 (0.664–1.418)	0.877
III	2.159 (1.630–2.862)	<0.001	1.259 (0.792–2.003)	0.330	2.143 (1.595–2.880)	<0.001	1.181 (0.732–1.906)	0.496
**T-stage**								
T1	Reference		Reference		Reference		Reference	
T2	1.047 (0.772–1.420)	0.766	0.928 (0.673–1.281)	0.650	1.190 (0.865–1.639)	0.285	1.058 (0.758–1.477)	0.742
T3	1.354 (0.996–1.839)	0.053	0.975 (0.675–1.407)	0.892	1.373 (0.994–1.896)	0.054	1.028 (0.704–1.501)	0.885
T4	1.828 (1.371–2.439)	<0.001	1.262 (0.851–1.871)	0.247	1.834 (1.352–2.487)	<0.001	1.365 (0.908–2.052)	0.135
**N-stage**								
N0	Reference		Reference		Reference		Reference	
N1	1.471 (1.226–1.765)	<0.001	1.340 (1.055–1.703)	0.017	1.608 (1.331–1.944)	<0.001	1.383 (1.078–1.773)	0.011
N2–3	1.839 (1.508–2.242)	<0.001	1.628 (1.215–2.180)	0.001	1.833 (1.492–2.250)	<0.001	1.670 (1.233–2.262)	0.001
**Radiotherapy**								
No/Unknown	Reference		Reference		Reference			
Yes	1.261 (1.054–1.507)	0.011	1.213 (0.994–1.481)	0.057	1.167 (0.970–1.405)	0.102		
**Chemotherapy**								
No/Unknown	Reference		Reference		Reference		Reference	
Yes	0.745 (0.641–0.867)	<0.001	0.513 (0.432–0.609)	<0.001	0.791 (0.676–0.925)	0.003	0.592 (0.500–0.701)	<0.001

^a^ OS, overall survival.

^b^ HR, hazard ratio.

^c^ CI, confidence interval.

^d^ Includes separated, single (never married), divorced, unmarried or domestic partners, unknown and widowed.

^e^ Grade was classified as well-differentiated (I), moderately differentiated (II), poorly differentiated (III), and undifferentiated (IV).

Multivariable analysis performed with the Cox regression model included laterality, age, gender, summary stage, grade, lymph node dissection, tumor stage, T-stage, N-stage, radiotherapy, and chemotherapy. The results showed that laterality, age, gender, grade, lymph node dissection, N-stage, radiotherapy, and chemotherapy were independent predictors of survival time in OS (*P* < 0.05; Table 2), with radiotherapy appearing as a negative prognostic factor with increased risk of death for OS (HR: 1.261; 95% CI 1.054–1.507; *P* = 0.011) and chemotherapy appearing as an independent predictor of improved for OS (HR: 0.745; 95% CI 0.641–0.867; *P* < 0.001).

### Propensity score matching survival analysis

All the variables were well balanced between the two groups after 1:1 PSM. The propensity scores before matching were 0.399 ± 0.082 for left PNs and 0.430 ± 0.089 for right PNs (*P* < 0.001). After matching, the propensity scores were 0.419 ± 0.085 for left PNs and 0.424 ± 0.084 for right PNs (*P* = 0.288). Finally, a total of 1,100 patients (550 with left PNs and 550 with right PNs) were included in our study. We found there were no significant differences in baseline characteristics between the matched groups (Table 1). The mean follow-up time was 31.78 ± 24.20 months (35.10 ± 25.18 months, left PNs and 28.46 ± 22.71 months, right PNs). Median OS was 35 (95% CI 29.44–40.56) months following a left PN, vs. 32 (95% CI 23.98–40.02) months following a right PN. One-, three-,and five-year OS rates for all patients, left and right PN patients were 72.5%, 47.7%, and 39.5%; 76.2%, 48.2%, and 39.1%; 68.8%, 47.4%, and 40.3%, respectively. Kaplan–Meier survival analysis implied between-group OS was not significantly different after matching (HR: 1.060; 95% CI 0.906–1.240; *P* = 0.465; Figure 2B).

### Subgroup analysis in matched groups

**Figure 4:**
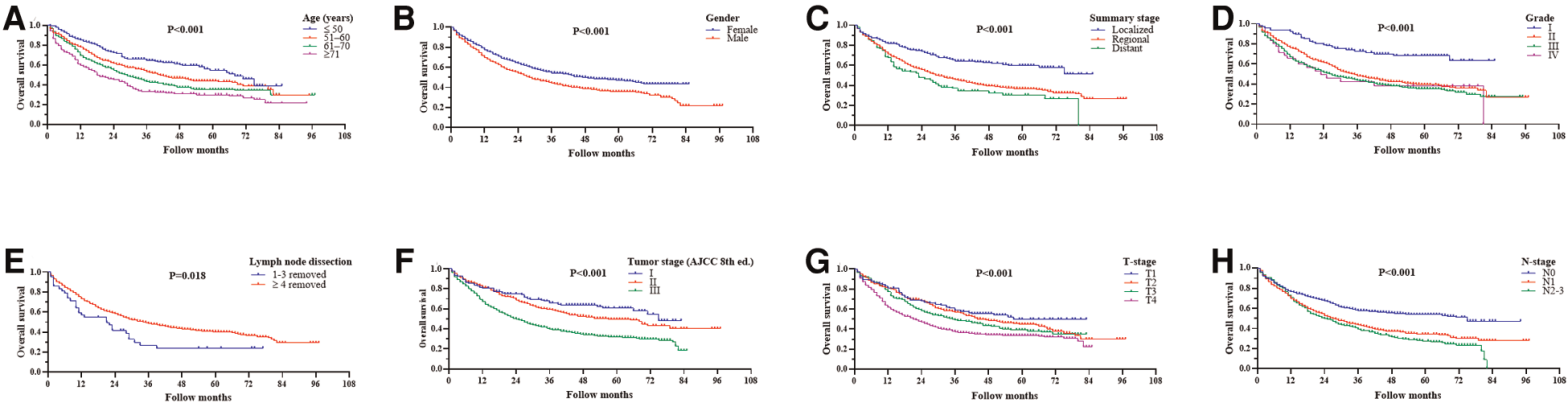
Ording to age (**A**), gender (**B**), summary stage (**C**), grade (**D**), lymph node dissection (**E**), tumor stage (**F**), T-stage (**G**), and N-stage (**H**).

**Figure 5:**
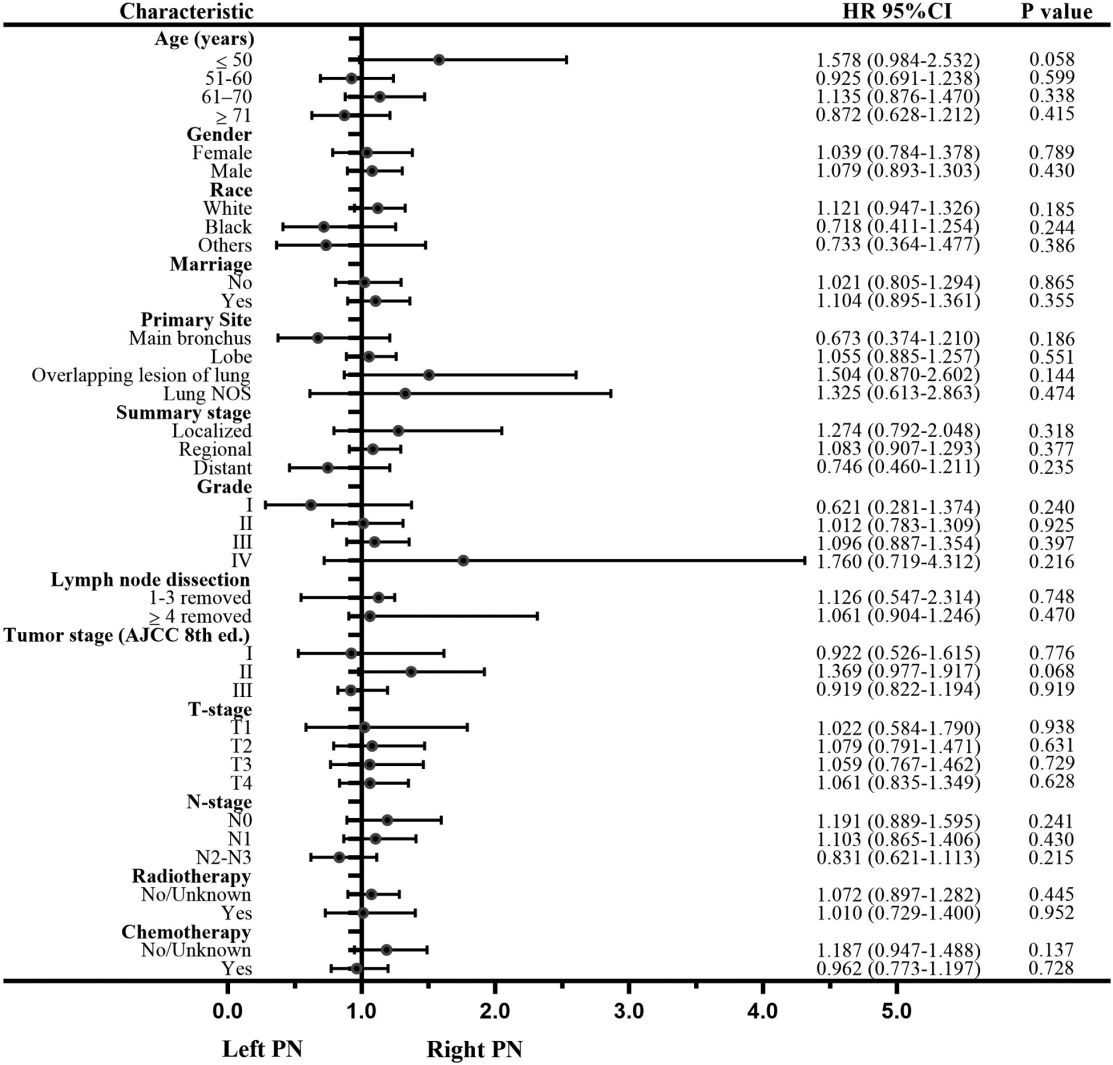
Forest plot of individual hazard ratios for the overall survival in left vs. right pneumonectomies.

Univariate analysis to identify possible prognostic factors after matching found statistically significant correlations between OS and age, gender, summary stage, grade, lymph node dissection, tumor stage, T-stage, N-stage, and chemotherapy (*P* < 0.05) (Figure 4). However, the results showed that radiotherapy was not an independent prognostic factor (HR: 1.167; 95% CI 0.970–1.405; *P* = 0.102; Figure 3D). The subsequent multivariable Cox regression model showed that age ≥51 (*P *≤ 0.005), male (*P* = 0.008), higher tumor grade (*P *≤ 0.004), <4 lymph node dissections removed, and higher N-stage (*P* < 0.05) were significant independent negative prognostic factors. The results also revealed that chemotherapy was an independent predictor of improved OS (HR: 0.791; 95% CI 0.676–0.925; *P* = 0.003; Figure 3B). The forest plot of individual HRs for OS in patients with left PNs vs. right PNs (Figure 5).

## Discussion

Anatomic surgical resection is currently the preferred method of treating lung cancer. Central tumors may be amenable to lobectomy or bronchial sleeve lobectomy (12, 13). With the advancement of technology, the wide application of high-resolution spiral computed tomography and the improvement of economic level, more lung cancer patients receive early surgical intervention. Some patients might receive direct radiotherapy and chemotherapy, target therapy or immunotherapy and other medical interventions but no PN treatment. In recent years, the clinical application of pneumonectomy has gradually decreased. But in patients with large tumors, tumor invasion of the left or right main bronchus and tumors crossing lung fissures, anatomic resection cannot be completed and a PN is required to achieve a clinical effect (14, 15). Nonetheless, PNs have relatively high morbidity and mortality (5.0%–10.0%) in the treatment of lung cancer (16). The operation is traumatic and the risk of postoperative complications including cardiac arrhythmias, cardiac failure, pulmonary infection, bronchopleural fistula, and acute respiratory distress syndrome (ARDS) is high (6, 7, 17, 18). Complication rates are generally higher after a right PN and these may affect long-term survival outcomes. However, due to the defects of the SEER database itself, we did not conduct further analysis. Martin et al. (19) had a total mortality rate of 3.8% after a PN (18/470), with an overall incidence of PNs of 38.1% (179/470). Ludwig et al. (20) reported that the 5-year OS rate after a PN was 27%, while Wang et al. (21) reported a post-PN 5-year survival rate of 46.3% in patients with pIII-N2 NSCLC. In this study, 1-, 3-, and 5-year OS rates after left and right PN were 82.0%, 58.4%, and 49.0%; and 67.6%, 46.3%, and 39.3%, respectively. Previously, PSM and OS were worse after right PNs vs. left PNs (HR: 1.459; 95% CI 1.254–1.697; *P* < 0.001). However, after matching in this study, 1-, 3-, and 5-year OS rates were 76.2%, 48.2%, and 39.1%; 68.8%, 47.4%, and 40.3%, respectively. Between-group OS was not significantly different after matching (HR: 1.060; 95% CI 0.906–1.240; *P* = 0.465). Yang et al. (22) reported similar findings, but they did not find a significant difference in the 5-year survival rate between left and right PNs before or after matching. We found that laterality did not affect survival. The reasons may be as follows: (1) there is a bias in patient selection, for example, patients with right PN are younger, have better lung function, and have no history of cardiovascular and cerebrovascular diseases; (2) for right PN patients in perioperative management more standardized.

In our study, several factors affecting PNs were analyzed. Generally speaking, the older the age, the greater the perioperative death and the worse the long-term survival prognosis (23). However, Bernet et al. found that age has nothing to do with long-term survival prognosis (24). Therefore, when a PN is required for elderly patients, the choice should be made cautiously in terms of postoperative oncology results and loss of physiological function. In this study, the long-term prognosis of male patients was poor. However, some scholars believe that gender is not a factor affecting prognosis (25).

Usually, the higher the degree of tumor differentiation, the worse the prognosis. Mediastinal lymph node metastasis is an unfavorable factor affecting the prognosis of lung cancer patients (26). We reported that relative N0, N1, and N2–3 had poor prognoses. Therefore, for patients undergoing a PN, strict staging should be performed before surgery, and methods such as PET-CT should be used to assess lymph node metastasis. In addition, the greater the number of lymph nodes dissected, the longer the OS may be; this is representative of the real-world situation (27). For patients with persistent hemoptysis, medical and interventional therapy are ineffective; or when intraoperative lobectomy and sleeve resection fail in patients with preoperative neoadjuvant therapy, PN is also required for lymph node advanced. In some cases, the stage can be lowered with neoadjuvant treatment before surgery (28, 29), but for patients whose tumors cannot be completely removed, PNs should be abandoned. Currently, neoadjuvant therapy is also controversial (30). More clinical trials are needed for further exploration in the future. It is generally believed that OS after a right PN is shorter than for a left PN. This may be related to the development of ARDS and bronchopleural fistulas after right PNs (22). Similar to a previous study, in the field of laterality (31), our study showed that after PSM, there is no difference in OS between the left and right PNs (*P* = 0.763).

Patients undergoing right PNs lose more lung capacity than those undergoing left PNs because the right lung accounts for 55%–60% of the total lung volume. Therefore, preoperative optimization of cardiopulmonary function before a right PN is particularly important. However, Deslauriers et al. (32) reported that expiratory lung function decreased by approximately 30% following a PN regardless of the operation side, indicating that even though the proportion of lung volume loss is greater after a right PN, long-term postoperative adjustments in pulmonary function may allow patients to adapt and lead near-normal lives. Ilonen et al. (33) also reported that there was no significant difference in pulmonary function after right vs. left PNs. Nonetheless, the relationship between lung function and survival prognosis after a PN remains controversial and there may be a poorer prognosis after a right PN vs. left PN. In this study, we did not compare the difference in lung function in relation to long-term survival after a PN because of the shortcomings of the database itself.

With the continuous advancement of thoracoscopy technology, which is more minimally invasive and leads to a more rapid recovery compared to traditional open surgery, a thoracoscopic lobectomy has better perioperative results and the same survival prognosis, such as fewer postoperative complications, less postoperative pain, aesthetics, and a shorter hospital stay. Whether thoracoscopic PNs will benefit patients remains unclear. Flores et al. (34) and Bendixen et al. (35) showed that there was no statistical difference between the two groups in long-term prognosis. Al Sawalhi et al. (36) reported that compared with open surgery, complications and oncologic outcomes were similar to uniportal video-assisted thoracoscopic surgery (VATS) PN. But shorter length of hospital stays, lower postoperative pain and superior OS with the uniportal VATS PN. Robotic PN has been gradually developed in recent years. The robotic approach offers many technical advantages, such as accurate identification of tissue planes, better optics, natural movement of the operator's hand is used to control the instrument for safe dissection and more ergonomic (37). May be better in long-term overall survival of PN. However, most are case reports (38–40) due to safety, surgical technique, and oncological factors, there are few single-center or multi-center reports of a series of robotic PN. In the future, multi-center randomized controlled trials should be carried out to further prove the advantages and safety of robotic PN.

This study was based on public data from the SEER database, even if we used PSM analysis, there could be bias and errors. Several limitations were found in this study: (1) The study lacked detailed information regarding complications, and adjuvant therapy (chemotherapy, radiotherapy, targeting, immunotherapy, etc.), regardless of whether it was pre- or post-operative. (2) There are data biases because we grouped the no or unknown variables into one group. (3) We used the 8th AJCC staging system, which replaced the 6th and 7th editions, leading to inconsistencies in the data transformation process. (4) The SEER database lacked information on tumor markers, smoking history, imaging, etc. and although our study did not address the impact of these factors on the prognosis in patients with PNs, they may play a significant role.

## Conclusion

There was no significant difference in long-term survival between patients with NSCLC undergoing a left vs. right PN. Laterality was not a prognostic factor for survival after a PN. Both neoadjuvant and adjuvant chemotherapy can prolong postoperative survival and either can be recommended if a PN is indicated. Additional long-term survival and outcomes analyses should be conducted on larger numbers of patients.

## Data Availability

The original contributions presented in the study are included in the article/**Supplementary Material**, further inquiries can be directed to the corresponding author.
